# Uncertainty analysis of model inputs in riverine water temperature simulations

**DOI:** 10.1038/s41598-021-99371-0

**Published:** 2021-10-07

**Authors:** Babak Abdi, Omid Bozorg-Haddad, Xuefeng Chu

**Affiliations:** 1 Department of Irrigation and Reclamation Engineering, Faculty of Agricultural Engineering and Technology, College of Agriculture and Natural Resources, University of Tehran, Karaj, Tehran Iran; 2 Department of Civil and Environmental Engineering, North Dakota State University, Dept 2470, Fargo, ND 58108-6050 USA

**Keywords:** Climate sciences, Ecology, Environmental sciences, Hydrology, Natural hazards, Solid Earth sciences

## Abstract

Simulation models are often affected by uncertainties that impress the modeling results. One of the important types of uncertainties is associated with the model input data. The main objective of this study is to investigate the uncertainties of inputs of the Heat-Flux (HFLUX) model. To do so, the Shuffled Complex Evolution Metropolis Uncertainty Algorithm (SCEM-UA), a Monte Carlo Markov Chain (MCMC) based method, is employed for the first time to assess the uncertainties of model inputs in riverine water temperature simulations. The performance of the SCEM-UA algorithm is further evaluated. In the application, the histograms of the selected inputs of the HFLUX model including the stream width, stream depth, percentage of shade, and streamflow were created and their uncertainties were analyzed. Comparison of the observed data and the simulations demonstrated the capability of the SCEM-UA algorithm in the assessment of the uncertainties associated with the model input data (the maximum relative error was 15%).

## Introduction

Mathematical modeling is subject to uncertainties from multiple sources, such as measurement errors and inputs, and model structure^19^. Each of these sources of uncertainties contributes to the generation of uncertain outputs and results from the modeling. Uncertainty analysis for the input data and model parameters can be performed by specifying the ranges of their variations and identifying the most probable values from these ranges. For example, in hydrologic modeling, uncertainties associated with the amount and timing of streamflow can be presented by incorporating the full range of simulation results of a hydrologic model^7^. Sun et al.^14^ evaluated the uncertainty of the Storm Water Management Model (SWMM) by using a generalized likelihood uncertainty estimation (GLUE). The GLUE based uncertainty analysis generated a probability distribution of possible outcomes from SWMM, which was compared with the Gaussian distribution developed by using the observed streamflow data. Feyen et al.^5^ used the GLUE algorithm to analyze the uncertainty of the hydraulic conductivity parameter in their MODFLOW modeling for a groundwater capture zone. They found that the delineated capture zones were most sensitive to the mean hydraulic conductivity. Zheng et al.^22^ analyzed the parameter uncertainty in an irrigated and rainfed agroecosystem model. They used the DayCent agroecosystem model and examined the role of parameter uncertainties in characterizing production functions. Their study indicated that incorporating rigorous estimates of uncertainty significantly enhanced the use of water production functions for effective water management. Yan et al.^16^ evaluated the impact of parameter uncertainty and water stress parameterization on wheat growth simulations by using the GLUE algorithm in the CERES-Wheat modeling. They found that GLUE-calibrated parameters were significantly different from the observations and concluded that this disagreement resulted mainly from the unrealistic water stress parameterization, which strongly affected the GLUE algorithm in selection of the calibrated parameters.

The Monte Carlo simulation has been used for uncertainty analysis as it provides multiple benefits over the conventional uncertainty analysis methods^13^. The Markov chain Monte Carlo (MCMC) combines the Monte Carlo approach with the Markov Chain to estimate the uncertainty bound of a model output. MCMC-based algorithms update the characteristics of the proposed probability distribution by maintaining the ergodicity^21^. Although the uncertainties associated with model parameters have been examined in numerous studies, the MCMC-based algorithms are able to account for input errors and/or model structural errors. Also, such algorithms use time-dependent parameters for exploring model deficits and considering the uncertainties of inputs and model structure^17^. Among the MCMC algorithms, the Shuffled Complex Evolution Metropolis algorithm (SCEM-UA) has been used in various hydrologic and environmental studies^20^. The SCEM-UA algorithm combines the strengths of the Metropolis algorithm, controlled random search, competitive evolution, and complex shuffling to infer the posterior target distribution by a continuous updating process^20^. Due to these advantages, the SCEM-UA algorithm was selected for the current study. Lin et al.^9^ used the GLUE algorithm and the SCEM-UA to address the issue of parameter uncertainty for conceptual hydrological modeling. Their results showed that when setting the threshold value at the interior sites, the simulated runoff series by the Xinanjiang model with the behavioral parameter sets fitted better with the observed runoff series. Liu et al.^10^ used the SCEM-UA to calibrate a hydrological model. They represented the posterior distribution functions of the hydrologic model output by the SCEM-UA. The calibration of the model, using 16 different parameters, was performed using two procedures of SCEM-UA and the genetic algorithm (GA). Results indicated that both methods were performing very well, but the SCEM-UA was better. Ajami et al.^3^ proposed the Integrated Bayesian Uncertainty Estimator (IBUNE) to analyze the uncertainty of model parameters. They used the SCEM algorithm to analyze the parameter uncertainty of a rainfall-runoff model. By utilizing the Bayesian model average (BMA) and the developed SCEM algorithm, they examined the total uncertainty of the model. Tang et al.^15^ used the SCEM-UA algorithm to analyze the uncertainties for nonlinear structural systems and demonstrated that the algorithm effectively estimated the parameters with uncertainties. Liu et al.^11^ evaluated the uncertainty of urban flood modeling. Due to the lack of reliable discharge data, they combined experimental data and modelling to characterize the floods and map the inundated areas in Xiamen Island, China.

Quantification of the uncertainties for river temperature models based on heat fluxes is considered in evaluating the effectiveness of ecological restoration alternatives. In ecological restoration studies such as aquatic green infrastructure^4^ and fish habitat^2^, small changes in driving parameters could cause faulty understanding from simulated water temperature, a fundamental factor affecting water quality and ecosystems (e.g.,^12,18^).

Thus, it is important to select the most efficient method to assess the uncertainties associated with the input data and model parameters, as most methods require longer running time and are computationally intensive for specific models. Neglecting uncertainty analysis can lead to a series of problems such as overdesign, higher costs, reduced reliability, and failure to achieve optimal benefits. Examining the uncertainties of model inputs provides a comprehensive insight into their influences on the model outputs, which potentially improves the modeling efficiency and lowers the cost in their measurements.

The objective of this study is to investigate the uncertainties of the inputs of the HFLUX model using the SCEM-UA algorithm. As a new effort, the SCEM-UA algorithm is used for assessing the HFLUX model inputs, and its performance is evaluated in this study. The HFLUX^8^ is an efficient and useful river water quality model for 1D simulation of the spatio-temporal distribution of stream water temperatures. In the simulation of temperature at each discretized node and each time step, the HFLUX considers the heat fluxes from the environment and lateral inflows of water to the node. It is also flexible in choosing the solution methods. Thus, the HFLUX model is selected for simulating water temperatures in this study. The simulation results are compared with the observed data to evaluate the performance of the SCEM-UA algorithm in the analysis of the uncertainties of the model inputs.

## Materials and methods

**Figure 1:**
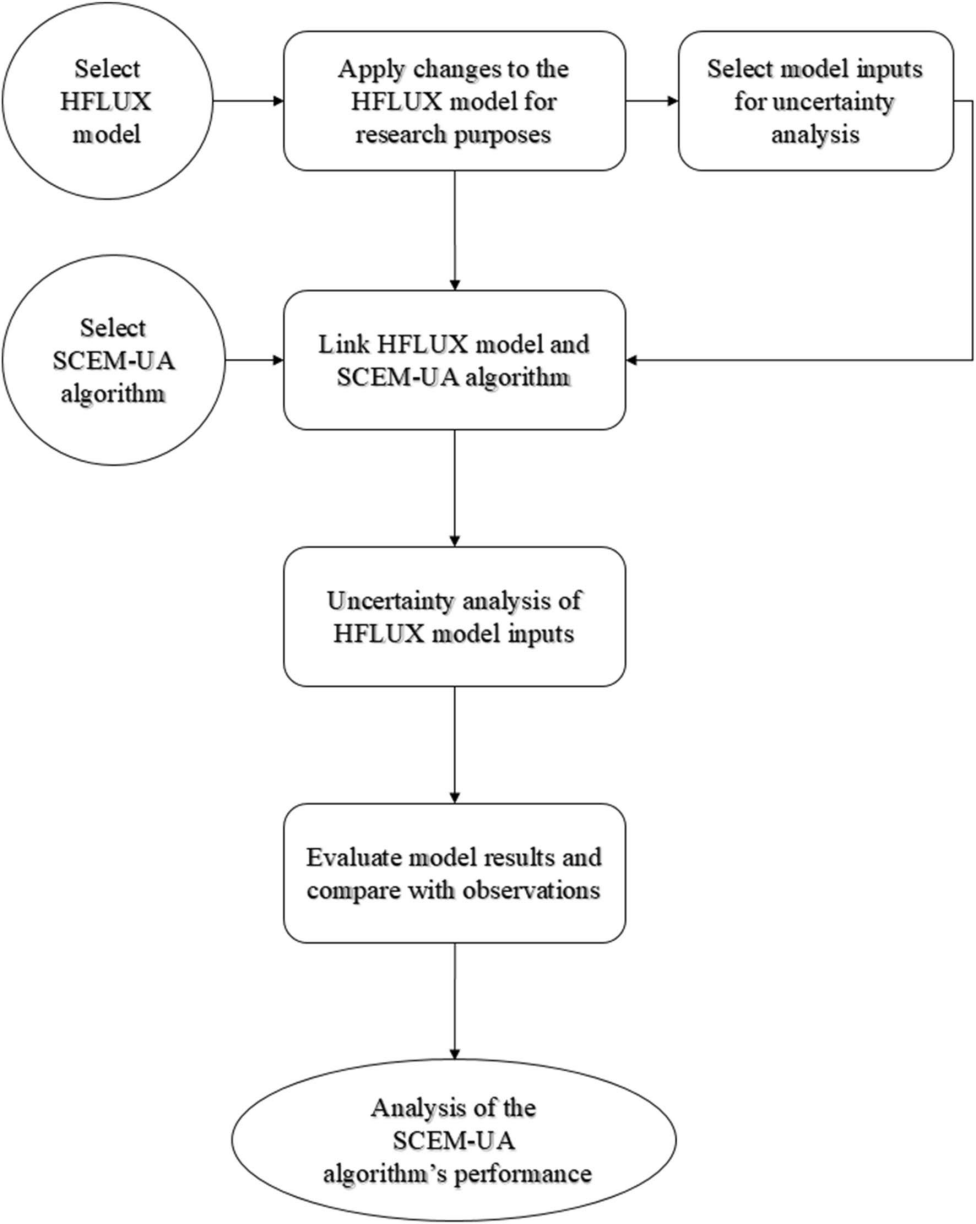
Flowchart for the uncertainty analysis.

In this study, the HFLUX model was coupled with the SCEM-UA algorithm for analyzing the uncertainties of the model inputs. The specific procedures started with selecting the inputs of the HFLUX model. With the linked HFLUX and SCEM-UA model and implementation of an iteration scheme, the uncertainty of each of the selected inputs was obtained based on the ranges (minimum and maximum values) of the input data/parameters and the Latin hypercube sampling. The simulations were then compared against the observed data to evaluate the performance of the SCEM-UA algorithm. These steps are depicted in Fig. 1.

### River water temperatures simulated by the HFLUX model

River water temperature affects the water quality and the ecosystem health, and hence control of river water temperature is important to mitigation of its adverse effects^1^. The HFLUX model was used to simulate the streamflow temperatures at different locations and times. The model is highly flexible in terms of choosing the solution methods for solving the governing equations and selecting the energy budget terms such as shortwave solar radiation, latent heat flux, and sensible heat transfer flux. The model input data include the initial spatial and temporal temperature conditions, stream geometry data, discharge data, and meteorological data^8^. The water balance and energy balance equations are respectively given by^8^:

1 $$\frac{\partial A}{{\partial t}} + \frac{\partial Q}{{\partial x}} = \mathop q\nolimits_{L}$$

2 $$\frac{{\partial \left( {A\mathop T\nolimits_{w} } \right)}}{\partial t} + \frac{{\partial \left( {Q\mathop T\nolimits_{w} } \right)}}{\partial x} = \mathop q\nolimits_{L} \mathop T\nolimits_{L} + R$$

3 $$R = \frac{{B\mathop \varphi \nolimits_{total} }}{{\mathop \rho \nolimits_{w} \mathop C\nolimits_{w} }}$$

where *A* is the cross section area of the stream (m^2^), *x* is the distance along the stream (m), *t* is the time (s), *Q* is the discharge of the stream (m^3^/s), *q*_*L*_ is the lateral inflow per unit stream length (m^2^/s), *T*_*w*_ is the stream temperature ($^\circ C$), *T*_*L*_ is the temperature of the lateral inflow ($^\circ C$), *R* is the energy flux (source or sink) per unit stream length ($^\circ C$ m^2^/s), *B* is the width of the stream (m), $\mathop \varphi \nolimits_{total}$ is the total energy flux to the stream per surface area (W/m^2^), $\mathop \rho \nolimits_{w}$ is the density of water (kg/m^3^), and $\mathop C\nolimits_{w}$ is the specific heat of water (J/kg $^\circ C$). Equation (3) is based on a thermal datum of 0 $^\circ C$ and the impact on the absolute value of the advective heat flux term. In Eq. (2), if *q*_*L*_ is negative, the first term on the right-hand side of the equation becomes a loss of *q*_*L*_*T*_*w*_. Also, dispersive heat transport that is omitted in Eq. 2 is negligible when the longitudinal change in water temperature is small in comparison to the temporal changes^8^.

### SCEM-UA algorithm

The SCEM-UA algorithm provides posterior distribution functions for the model parameters and input data by generating an initial sample from the parameter space. First, the indicators of *n*, *q*, and *s* that are respectively dimension (the number of investigate inputs), number of complexes (the population to be divided), and population (the number of sample points) are determined for the algorithm. Then, the algorithm searches the sampling points in the feasible space and sorts the points according to the density. The algorithm determines the sequence and complexes based on those points. The sequence is the first *q* points of the population and complexes are a collection of *m* points from the population. Note that *m* = *s*/*q*. In the next step, the points of each complex are sorted based on the density, which can be mathematically expressed as^20^:

4 $$\left\{ {\begin{array}{*{20}c} {\mathop \alpha \nolimits^{k} \le T\,\,\,\,\,\,\,\,\,\mathop \theta \nolimits^{t + 1} = N\left( {\mathop \theta \nolimits^{t} ,\,\mathop C\nolimits_{n}^{2} \mathop \Sigma \nolimits^{k} } \right)} \\ {\mathop \alpha \nolimits^{k} > T\,\,\,\,\,\,\,\,\mathop \theta \nolimits^{t + 1} = N\left( {\mathop \mu \nolimits^{k} ,\,\mathop C\nolimits_{n}^{2} \mathop \Sigma \nolimits^{k} } \right)} \\ \end{array} } \right.$$

where *k* = 1,2,…,*q*, *α* is the ratio of the mean posterior density of the *m* points of complexes to the mean posterior density of the last *m* generated points of sequences, $\theta$ is the points of complexes, ${c}_{n}=\frac{2.4}{\sqrt{n}}$ , $T={10}^{6}$, $\mu$ is the mean, and ∑ denotes the covariance. To investigate the new points created by the algorithm, the points of complexes are replaced by^20^:

5 $$\left\{ {\begin{array}{*{20}l} {\Omega \ge Z\quad replace\,best\,member\,of\,\mathop C\nolimits^{k} \,with\,\mathop \theta \nolimits^{t + 1} } \\ {\Omega < Z\quad \mathop \theta \nolimits^{t + 1} = \mathop \theta \nolimits^{t} \,\,\,\,\,\,\,\,\,\,\,\,\,\,\,\,\,\,\,\,\,} \\ \end{array} } \right.$$

where $\mathop C\nolimits^{k}$ is the *K*^th^ complex, *Z* is drawn from the uniform distribution in the range of 0–1, and Ω is calculated by^20^:

6 $$\Omega = \frac{{P\left( {\left. {\mathop \theta \nolimits^{t + 1} } \right|y} \right)}}{{P\left( {\left. {\mathop \theta \nolimits^{t} } \right|y} \right)}}$$

where $P\left( {\left. {\mathop \theta \nolimits^{t + 1} } \right|y} \right)$ and $P\left( {\left. {\mathop \theta \nolimits^{t} } \right|y} \right)$ are the posterior probability distributions for $\mathop \theta \nolimits^{t + 1}$ and $\mathop \theta \nolimits^{t}$, respectively. Then, the algorithm examines the following condition for each complex. If it is rejected, the algorithm replaces the worst member ${c}^{k}$(the point with the lowest density) with ${\theta }^{t+1}$ ^20^.

7 $$\mathop \Gamma \nolimits^{k} \le T\,\,and\,\,P\left( {{{\mathop \theta \nolimits^{t + 1} } \mathord{\left/ {\vphantom {{\mathop \theta \nolimits^{t + 1} } y}} \right. \kern-\nulldelimiterspace} y}} \right) < \,P\left( {{{\mathop C\nolimits_{m}^{k} } \mathord{\left/ {\vphantom {{\mathop C\nolimits_{m}^{k} } y}} \right. \kern-\nulldelimiterspace} y}} \right)$$

where ${\Gamma }^{k}$ is the ratio of the posterior density of the best (the point with the highest density) to the posterior density of the worst member of ${c}^{k}$. The last step is to examine $\beta$ and *L.* Note that $\beta$ = 1 and *L* = *m*/10. If $\beta < L$, $\beta = \beta + 1$ and the algorithm returns to sort complex points. Otherwise, the algorithm examines the Gelman and Rubin convergence^6^, and eventually provides the posterior distribution functions^20^. The value of the Gelman and Rubin convergence should be less than 1.2. The Gelman and Rubin convergence is examined by:

8 $$R = \sqrt {\frac{g - 1}{g} + \frac{q + 1}{{q.g}}\frac{B}{W}}$$

where *g* is the number of iterations within each sequence, *B* is the variance between the *q* sequence means, and *W* is the average of the *q* within-sequence variances for the parameter under consideration^20^.

### Study AREA

**Figure 2:**
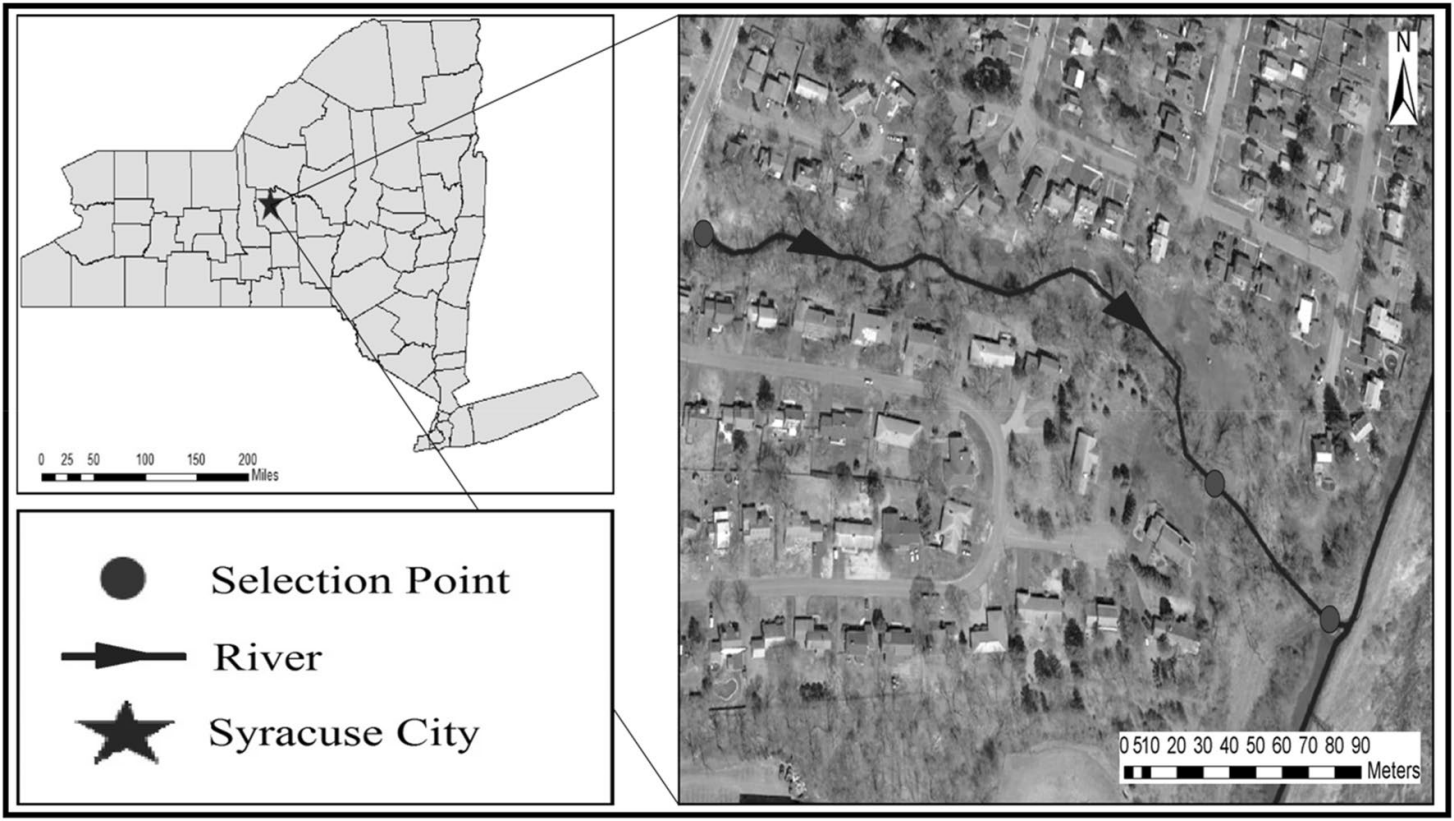
Study area and the locations of three evaluation sections (the gray enlarged map shows the State of New York), the map in this Figure is created by Google Earth 7.0.2.8415 (https://google.com/earth/versions).

Meadowbrook Creek was selected to test the methods proposed in this study^8^. The creek flows through the City of Syracuse in New York. Thus, this catchment consists of high residential and industrial land covers, which contribute runoff to the main channel. The creek is about 4 km long. A portion of this creek (475 m long) was selected for the modeling for a period of June 13–19, 2012 in this study. The upstream boundary condition in the HFLUX model was set based on the water temperature of the creek observed at the upstream station^8^. The uncertainty of the model inputs was examined at three selected points as shown in Fig. 2. Note that the input values at these three points had greater relative changes than the changes at other locations, which provided the possibility to improve the evaluation of the algorithm performance. In addition, these three locations had the same sampling of the selected input data. During the simulation period, the streamflow velocity varied within a range of 0.06–0.63 (m/s). The daily temperature changed between 8.9 and 28.2 °C. The relative humidity, used to calculate the total energy flux to the stream per surface area, changed from 36 to 93%. The creek bed mainly consisted of clay, cobbles, sand, and gravel materials. The basic statistics of the data/variables used in the HFLUX model are presented in Table 1. Figure 2 shows the study area, the creek, and the three selected points for analysis.

**Table 1:** Basic statistics of the data/variables used in the HFLUX model.

Variable	Mean	Std	Min	25%	50%	75%	Max
Water temperature at the upstream boundary (°C)	16.98	1.61	13.68	15.87	16.88	18.19	20.26
Discharge (m^3^/s)	0.0670	0.0039	0.0603	0.0634	0.0672	0.0705	0.0734
Air temperature (°C)	20.08	5.10	8.90	16.40	20.50	24.10	28.20
Relative humidity (%)	64.89	16.30	36.00	50.00	65.00	77.00	93.00
Shortwave solar radiation (W/m^2^)	251.23	364.63	0.00	0.00	24.00	469.36	1208.00

### Ethical approval

All authors accept all ethical approvals.

### Consent to participate

All authors consent to participate.

### Consent to publish

All authors consent to publish.

## Results and discussion

**Figure 3:**
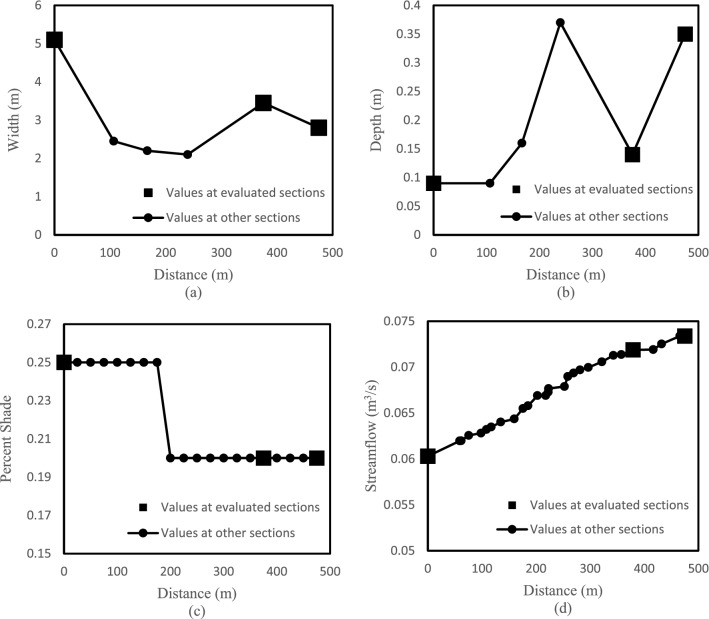
Characteristics of Meadowbrook Creek: (**a**) width, (**b**) depth, (**c**) percent shade, and (**d**) streamflow.

In the uncertainty analysis, the inputs at the three selected points on the main channel include: the depth and width of the creek, the percentage of shade, and the streamflow. Note that the shade value at a location ranges from 0 to 1 with 0 being no shade and 1 being total shade. The values of these inputs estimated by the SCEM-UA algorithm at different locations along the creek are shown in Fig. 3.

To implement the SCEM-UA, the posterior distribution functions for the selected inputs of the model were first developed. The initial ranges of the four selected inputs, according to the related literature and field observations, were selected to form the first generation of the SCEM-UA population using the Latin hypercube sampling. Table 2 presents the values of the maximum and minimum values of these inputs.

**Table 2:** The selected HFLUX inputs and their upper and lower limits considered in the uncertainty analysis.

Input	Unit	Minimum	Maximum	Description
*W*_1_	m	1	7	Creek width at the beginning of the study reach (0 m)
*W*_2_	m	1	7	Creek width at 375 m from the beginning of the study reach
*W*_3_	m	1	7	Creek width at the end of the study reach (475 m)
*D*_1_	m	0.001	1	Creek depth at the beginning of the study reach (0 m)
*D*_2_	m	0.001	1	Creek depth at 375 m from the beginning of the study reach
*D*_3_	m	0.001	1	Creek depth at the end of the study reach (475 m)
*Sh*_1_	-	0	1	Percent shade coefficient at the beginning of the study reach (0 m)
*Sh*_2_	-	0	1	Percent shade coefficient at 375 m from the beginning of the study reach
*Sh*_3_	-	0	1	Percent shade coefficient at the end of the study reach (475 m)
*Q*_1_	m^3^/s	0.05	0.1	Streamflow at the beginning of the study reach (0 m)
*Q*_2_	m^3^/s	0.05	0.1	Streamflow at 375 m from the beginning of the study reach
*Q*_3_	m^3^/s	0.05	0.1	Streamflow at the end of the study reach (475 m)

**Figure 4:**
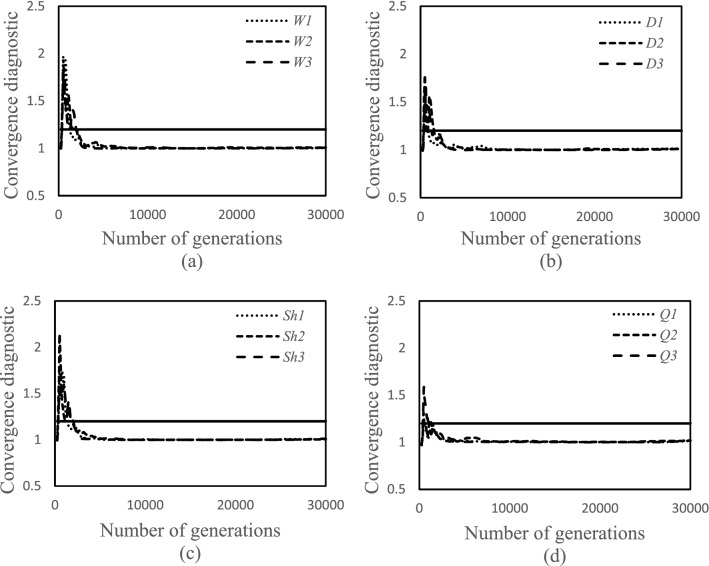
Gelman and Rubin convergence criterion chart: (**a**) width, (**b**) depth, (**c**) percent shade, and (**d**) streamflow.

The SCEM-UA simulations were performed for a spatial interval of 10 m and a time step of 30 min. The number of the SCEM-UA iterations considered for this study was 30,000, depending upon the Gelman and Rubin convergence criterion^6^, a statistical indicator used for examining the convergence of the chains. The value should be less than 1.2^20^. Figure 4 shows the changes in the Gelman and Rubin convergence, indicating that the values for all inputs are less than 1.2.

**Figure 5:**
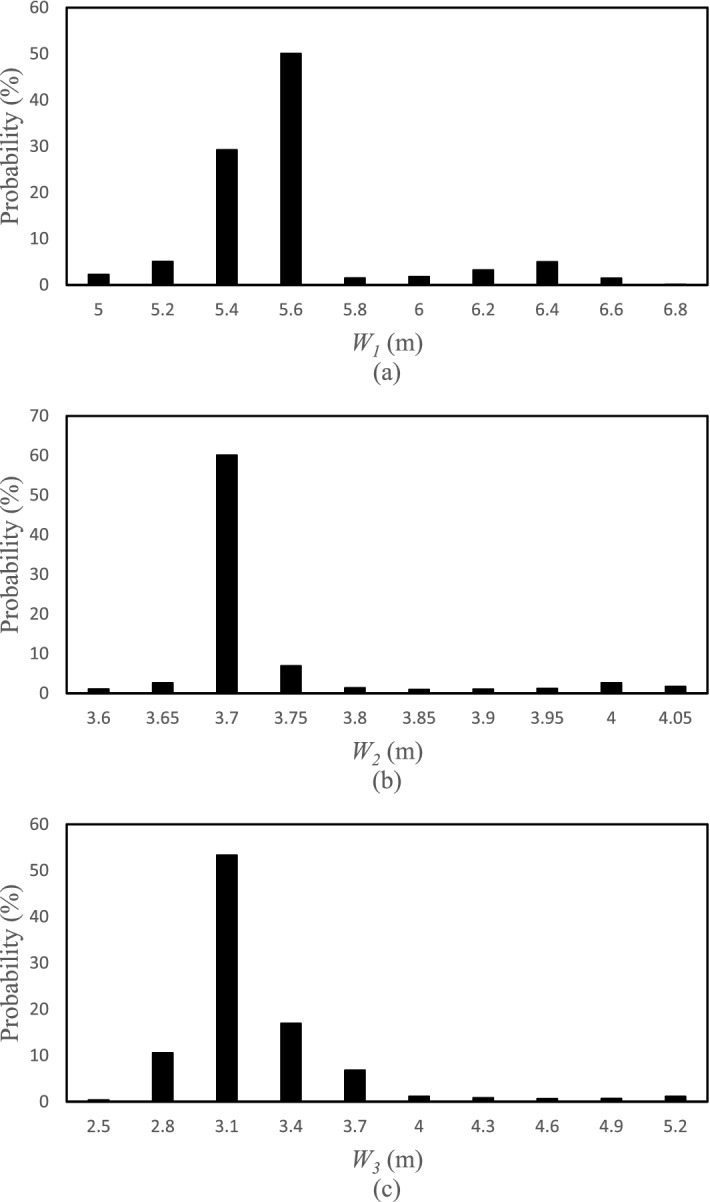
Histograms of the HFLUX inputs at three sections (**a**) Creek width at the beginning of the reach study range (0 m), (**b**) Creek width at 375 m from the beginning of the reach study range, and (**c**) Creek width at the end of the reach study range (475 m).

**Figure 6:**
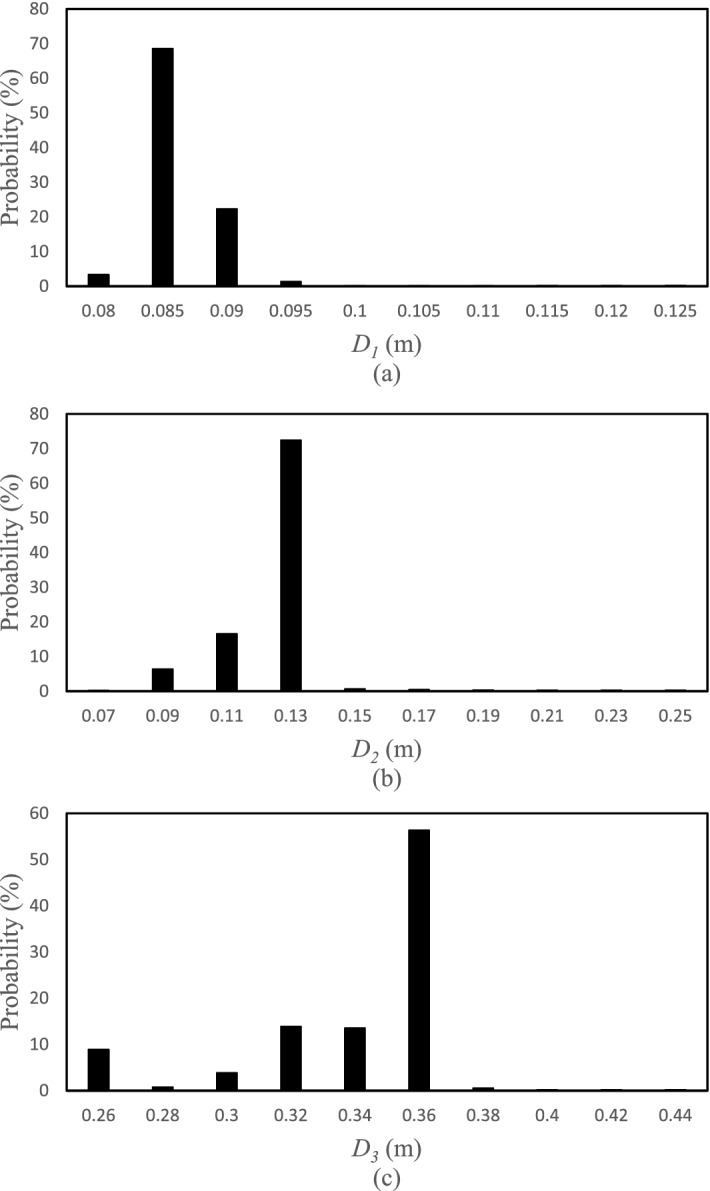
Histograms of the HFLUX inputs at three sections (**a**) Creek depth at the beginning of the reach study range (0 m), (**b**) Creek depth at 375 m from the beginning of the reach study range, and (**c**) Creek depth at the end of the reach study range (475 m).

**Figure 7:**
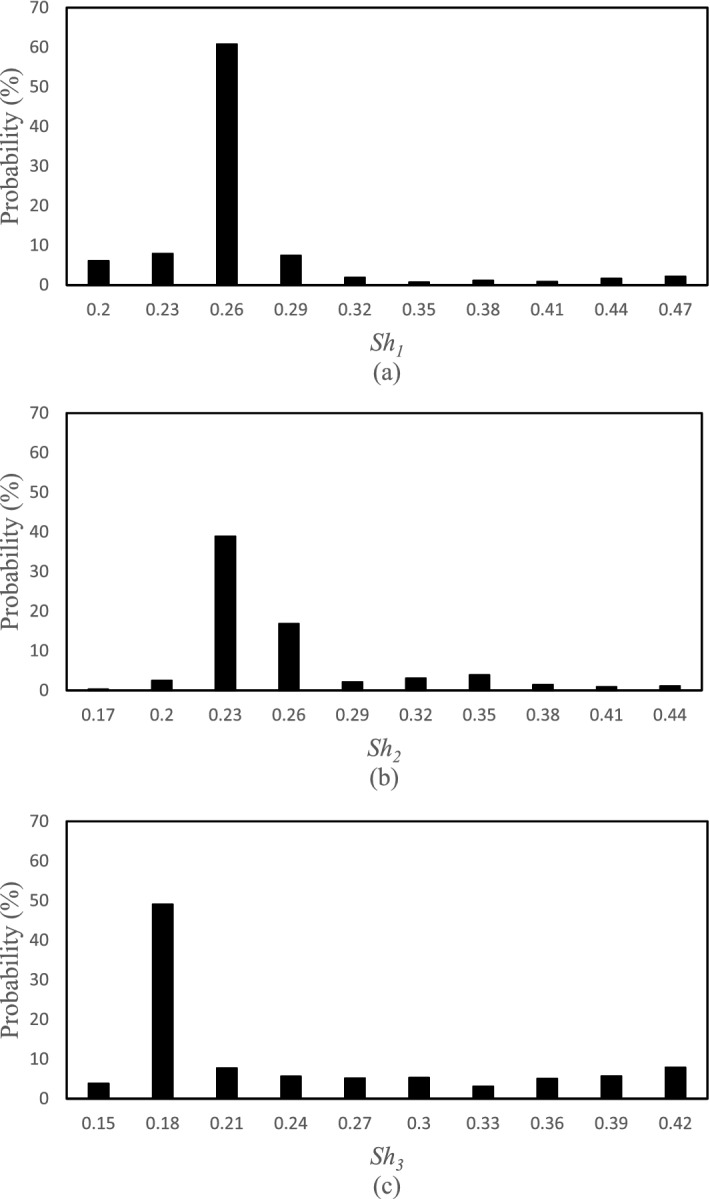
Histograms of the HFLUX inputs at three sections (**a**) percent shade coefficient at the beginning of the reach study range (0 m), (**b**) percent shade coefficient at 375 m from the beginning of the reach study range, and (**c**) percent shade coefficient at the end of the reach study range (475 m).

**Figure 8:**
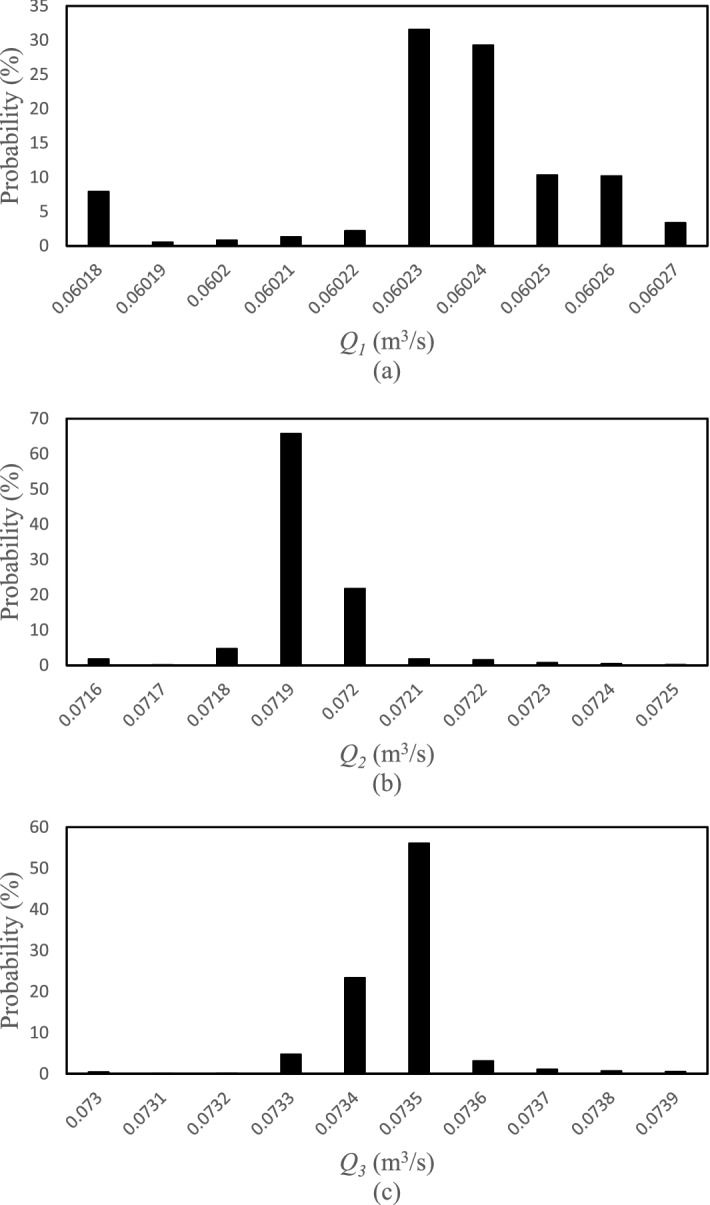
Histograms of the HFLUX inputs at three sections (**a**) streamflow at the beginning of the reach study range (0 m), (**b**) streamflow at 375 m from the beginning of the reach study range, and (**c**) streamflow at the end of the reach study range (475 m).

The posterior distribution functions for the selected points of the HFLUX model were obtained based on the SCEM-UA algorithm in the form of histograms for the inputs. The width or range of the histograms indicates the uncertainty of the inputs and the average value or the most likely value of the histograms is the most likely prediction value of the SCEM-UA algorithm for that input. Figures 5, 6, 7 and 8 show the histograms of the posterior distribution functions of the selected inputs. As shown in Fig. 5, the uncertainty for the creek width at the first point/location ranges from 5 to 6 and the most likely value predicted by the SCEM-UA is 5.6 with a probability of 50%. The range of the uncertainty for this input at the second point/location is from 3.6 to 4.05 and the most likely value is 3.7 with a probability of approximately 60%. For this input at the third point/location, the uncertainty ranges from 2.5 to 5.2 and the most likely value is 3.1 with a probability of 50%. According to Fig. 6, the uncertainty ranges for the creek depth at the first, second, and third points/locations are 0.08–0.125, 0.07–0.25, and 0.26–0.44, respectively. The most likely values for this input at the first, second, and third points/locations are 0.085 with a probability of nearly 70%, 0.13 with a probability of almost 70%, and 0.36 with a probability of almost 55%, respectively. Figure 7 shows the uncertainty ranges and the most likely values for the percentage of shade at the first, second, and third points/locations. The ranges for the tree points are respectively 0.2–0.47, 0.17–0.44, and 0.15–0.42, while the most likely values are respectively 0.26 with a probability of almost 60%, 0.23 with a probability of almost 40%, and 0.18 with a probability of almost 50%. Similarly, Fig. 8 shows the uncertainty ranges and the most likely values for the streamflow at the first, second, and third points/locations. The ranges for the three points respectively are 0.06018–0.06027, 0.0716–0.0725, and 0.073–0.0739, while the most likely values respectively are 0.06023 with a probability of almost 30%, 0.0719 with a probability of almost 60%, and 0.0735 with a probability of almost 55%.

**Figure 9:**
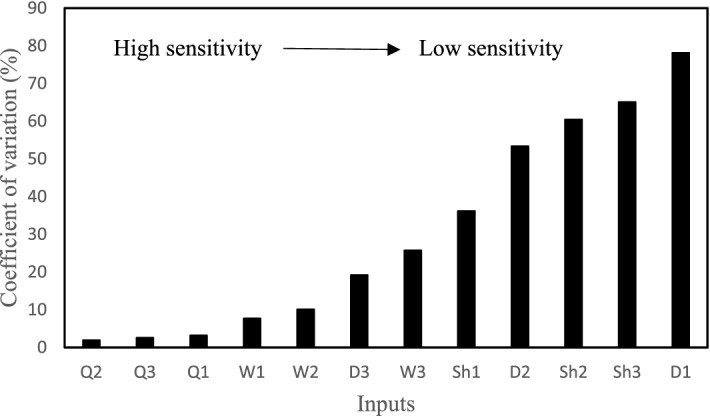
Order of sensitivity of the inputs quantified by the CV values.

**Figure 10:**
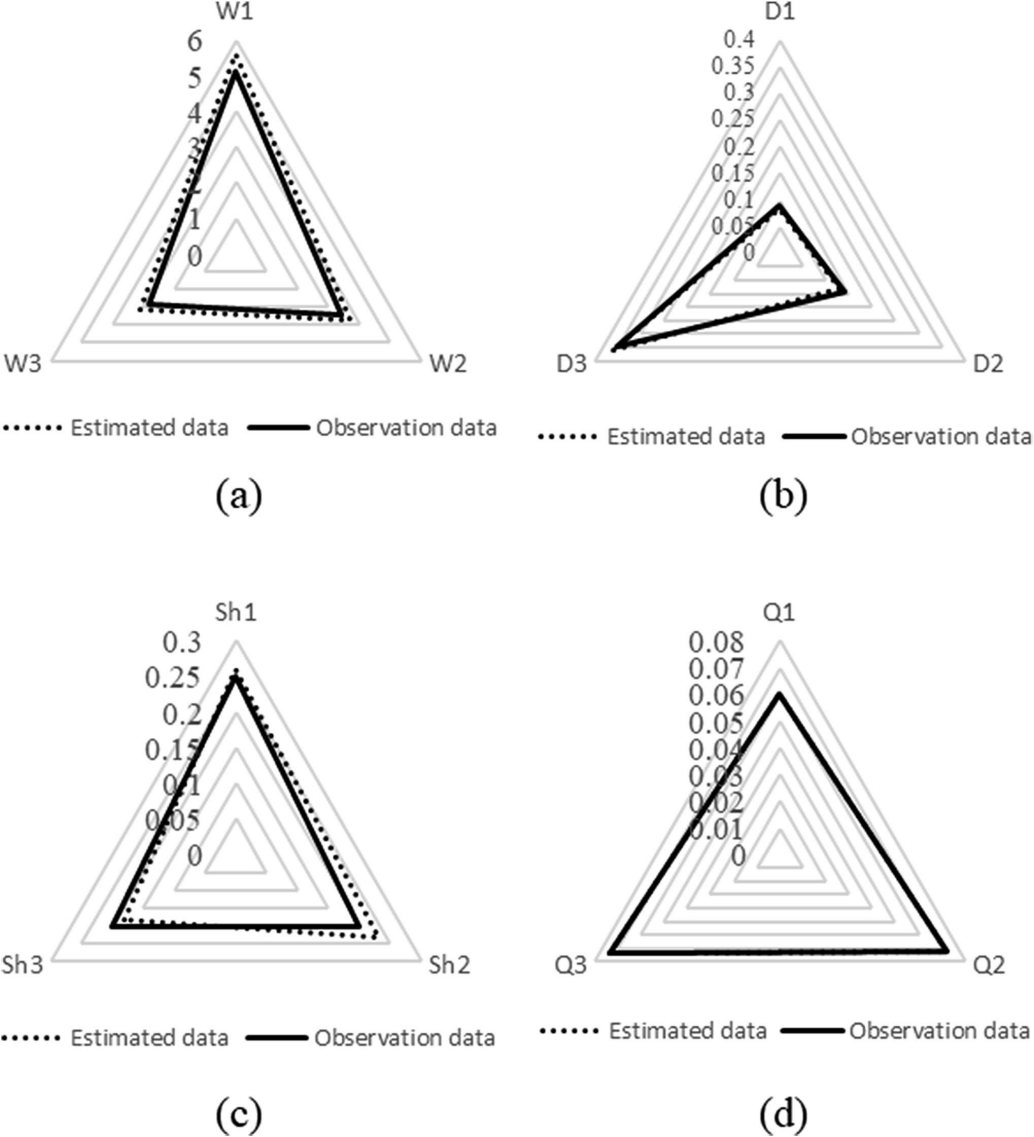
Comparison of the observed data and the most likely values estimated by the SCEM-UA algorithm: (**a**) width, (**b**) depth, (**c**) percent shade, and (**d**) streamflow.

Based on the statistical results related to the inputs, their sensitivity can be identified. The smaller the coefficient of variation (CV) of an input, the more sensitive the input. Figure 9 indicates the order of sensitivity of the inputs. Accordingly, inputs *Q*_2_, *Q*_3_, and *Q*_1_ with CV values of 1.94%, 2.57%, and 3.18%, respectively, have the higher sensitivity, implying very small changes in the histograms of these three points. Inputs *D*_1_, *Sh*_3_, and *Sh*_2_ with CV values of 78.14%, 65.08%, and 60.48%, respectively, have the lower sensitivity. Table 3 shows the CV and the most likely values of all inputs. Knowing the most likely value of each input is important in evaluating the performance of the uncertainty analysis algorithm. Figure 10 shows the comparison of the observed data and the most likely values estimated by the SCEM-UA algorithm. According to Fig. 10 the SCEM-UA algorithm overestimated the inputs of *W*_1_, *W*_2_, *W*_3_, *D*_3_, *Sh*_1_, *Sh*_2_, *Q*_2_, and *Q*_3_ and underestimated the inputs of *D*_1_, *D*_2_, *Sh*_3_, and *Q*_1_. The most likely values of the selected inputs simulated by the SCEM-UA algorithm were compared with the observed data and their relative errors are shown in Table 4. It can be observed that the maximum relative error (15%) is related to the percentage of shade for the second point/location. Thus, it is suitable to use the SCEM-UA algorithm for evaluating the uncertainties of the HFLUX inputs.

**Table 3:** Coefficient of variation (CV) and most likely values of inputs.

Input	CV (%)	Most likely value	Input	CV (%)	Most likely value
*W*1 (m)	7.71	5.6	*Sh*1 (dimensionless)	36.20	0.26
*W*2 (m)	10.10	3.7	*Sh*2 (dimensionless)	60.46	0.23
*W*3 (m)	25.74	3.1	*Sh*3 (dimensionless)	65.08	0.18
*D*1 (m)	78.14	0.085	*Q*1 (m^3^/s)	3.18	0.06023
*D*2 (m)	53.35	0.130	*Q*2 (m^3^/s)	1.94	0.07190
*D*3 (m)	19.19	0.360	*Q*3 (m^3^/s)	2.57	0.07350

**Table 4:** Relative errors of the inputs in the SCEM-UA algorithm.

Input	Relative error (%)	Input	Relative error (%)
*W*_1_	9.80	*Sh*_1_	4.00
*W*_2_	7.24	*Sh*_2_	15.00
*W*_3_	10.71	*Sh*_3_	10.00
*D*_1_	5.56	*Q*_1_	0.11
*D*_2_	7.14	*Q*_2_	0.03
*D*_3_	2.86	*Q*_3_	0.16

## Concluding remarks

This study focused on investigating the uncertainties of the HFLUX model inputs by using the SCEM-UA algorithm. Meadowbrook Creek in the City of Syracuse, New York was selected as an application of the proposed methods. The histograms of the selected model inputs were obtained, based on which the uncertainty of the inputs and their most likely values were determined. Specifically, the width of each histogram indicated the uncertainty of the corresponding input. It was found that the creek depth at the beginning of the study reach with a CV of 78.14% was the most uncertain and thus the least sensitive input. The streamflow at 375 m from the beginning of the study reach with a CV of 1.94% was the least uncertain and thus the most sensitive input. The mean of each histogram indicated the most likely value for the corresponding input. The performance of the SCEM-UA algorithm was evaluated by comparing the observed data and the most likely values from the SCEM-UA algorithm. Based on the comparisons, the streamflow at 375 m from the beginning of the study reach with the smallest relative error (0.03%) was the most accurately estimated input, while the percent shade coefficient at 375 m from the beginning of the study reach with the largest relative error (15%) was the least accurately estimated input. These results demonstrated that the SCEM-UA algorithm was suitable for analyzing the uncertainties associated with the inputs of the HFLUX model.

## Data Availability

All of the required data have been presented in our article.
